# RNAi Therapeutics: How Likely, How Soon?

**DOI:** 10.1371/journal.pbio.0020028

**Published:** 2004-01-20

**Authors:** Richard Robinson

## Abstract

RNA interference (RNAi) is used in the lab to silence virtually any gene. Previously, antisense and ribozymes were successful in the lab, but have been disappointing in the clinic. Will RNAi succeed where these two have not?

RNA interference (RNAi) has been called “one of the most has exciting discoveries in biology in the last couple decades,” and since it was first recognized by Andrew Fire et al. in 1998, it has quickly become one of the most powerful and indispensable tools in the molecular biologist's toolkit. Using short double-stranded RNA (dsRNA) molecules, RNAi can selectively silence essentially any gene in the genome. It is an ancient mechanism of gene regulation, found in eukaryotes as diverse as yeast and mammals, and probably plays a central role in controlling gene expression in all eukaryotes. In the lab, RNAi is routinely used to reveal the genetic secrets of development, intracellular signaling, cancer, infection, and a full range of other phenomena. But can the phenomenon hailed by the journal *Science* as the “Breakthrough of the Year” in 2002 break out of the lab and lead to novel therapies as well? Pharmaceutical giants are hoping so, and several biotech companies have bet their futures on it, but not everyone is sanguine about the future of RNAi therapy.

At the heart of its promise as a powerful therapeutic drug lies the exquisite selectivity of RNAi: like the fabled “magic bullet,” an RNAi sequence seeks out and destroys its target without affecting other genes. The clinical applications appear endless: any gene whose expression contributes to disease is a potential target, from viral genes to oncogenes to genes responsible for heart disease, Alzheimer's disease, diabetes, and more.

But for all its promise, RNAi therapy is a long way from entering the clinic. While it is a proven wunderkind in the lab, to date no tests have been done in humans, and only the most modest and circumscribed successes have been demonstrated in animals. The road to clinical success is littered with great ideas that have come a cropper along the way, including two other RNA-based therapies, antisense and ribozymes, both of which showed promise at the bench but have largely stumbled before reaching the bedside. Is RNAi also likely to fall short? Or is it different enough to make this third try the charm?

## Clinical Naïveté, Mysterious Mechanisms

To be a successful drug, a molecule must overcome a long set of hurdles. First, it must be able to be manufactured at reasonable cost and administered safely and conveniently. Then, and even more importantly, it must be stable enough to reach its target cells before it is degraded or excreted; it must get into those cells, link up with its intracellular target, and exert its effect; and it must exert enough of an effect to improve the health of the person taking it. And, finally, it must do all this without causing significant toxic effects in either target or nontarget tissues. No matter how good a compound looks in the lab, if it fails to clear any one of these hurdles, it is useless as a drug.

**Figure 1:**
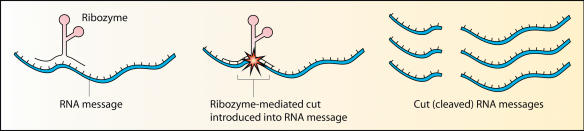
Ribozymes A ribozyme binds to a specific mRNA, cleaves it, and thus prevents it from functioning.

For RNA-based therapies, manufacture has been seen as a soluble problem, while delivery, stability, and potency have been the most significant obstacles. “There was a lot of clinical naïveté” in the early days of antisense and ribozymes, according to Nassim Usman, Vice President for Research and Development at Sirna Therapeutics in Boulder, Colorado. “Compounds were pushed into the clinic prematurely.” Sirna began as the biotech startup Ribozyme Pharmaceuticals, which tried to develop ribozymes to treat several conditions, including hepatitis C. A ribozyme is an RNA molecule whose sequence and structure allow it to cleave specific target RNA molecules (see Figure 1). “The initial results with hepatitis C were not that inspiring,” says Usman, because the molecule they used had low potency and a short half-life once in the body. Despite “enormous doses,” the viral load was not significantly affected. “It just didn't have the characteristics to be a drug,” he says. No ribozyme has yet been approved for use by the United States Food and Drug Administration (FDA).

**Figure 2:**
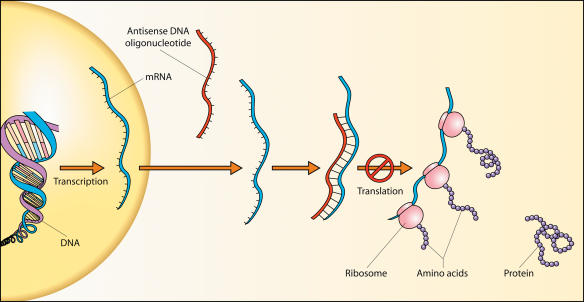
Antisense Antisense DNA or RNA binds to a specific mRNA and prevents it from being translated into protein.

Similarly, despite much initial enthusiasm, attempts to develop antisense drugs have been largely disappointing. Antisense is a single strand of RNA or DNA, complementary to a target messenger RNA (mRNA) sequence; by pairing up with it, the antisense strand prevents translation of the mRNA (see Figure 2). At least that was the theory, and early clinical results seemed to support the theory: antisense drugs effectively reduced tumor sizes in anticancer trials and viral loads in antiviral trials. But closer inspection revealed these results were largely due to an increase in production of interferons by the immune system in response to high doses of the foreign RNA, rather than to specific silencing of any target genes. (The relatively high proportion of C–G sequences in antisense mimics bacterial and viral genes, thus triggering the immune response.) When the antisense dose was lowered to prevent the interferon response, the clinical benefit largely disappeared as well. Thus, rather than being a highly specific therapy, antisense seemed to be a general immune system booster.

But as long as patients were getting better, does it matter what the mechanism was? “It doesn't matter if you are a patient, but it does matter if you are trying to develop the next drug,” says Cy Stein, Associate Professor of Medicine and Pharmacology at Columbia University College of Physicians and Surgeons in New York City. Stein has researched antisense for more than a decade. “If you get the mechanism wrong, you're not going to be able to do it.”

To date, only one antisense drug has received FDA approval. Vitravene, from Isis Pharmaceuticals in Carlsbad, California, is used to treat cytomegalovirus infections in the eye for patients with HIV. Vitravene is actually a DNA antisense drug, which binds to viral DNA, though, says Usman, “it's unclear whether it actually works by an antisense mechanism.” Stein expresses a similar skepticism about the mechanism of a second antisense drug, Genasense. Genasense is a DNA-based treatment that targets Bcl-2, a protein expressed in high levels in cancer cells, which is thought to protect them from standard chemotherapy. The FDA is currently reviewing an application for Genasense, based on promising results in the treatment of malignant melanoma.

## RNAi: A Natural Alternative

**Figure 3:**
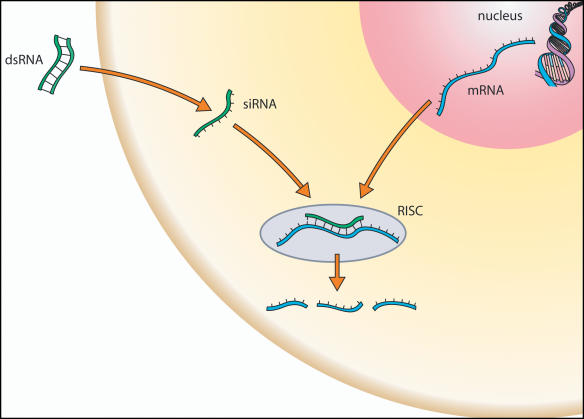
RNAi RNAi is a recently described naturally occurring process in which small RNA molecules activate a cellular process that results in the destruction of a specific mRNA.

Growing disillusionment with antisense and ribozymes coincided with the discovery of RNAi and the realization that it was a far more potent way to silence gene expression. RNAi uses short dsRNA molecules whose sequence matches that of the gene of interest. Once in a cell, a dsRNA molecule is cleaved into segments approximately 22 nucleotides long, called short interfering RNAs (siRNAs) (see Figure 3). siRNAs become bound to the RNA-induced silencing complex (RISC), which then also binds any matching mRNA sequence. Once this occurs, the mRNA is degraded, effectively silencing the gene from which it came. (Details of the structure and function of the RISC are still largely unknown, but it is thought to act as a true enzyme complex, requiring only one or several siRNA molecules to degrade many times that number of matching mRNAs.)

The extraordinary selectivity of RNAi, combined with its potency—in theory, only a few dsRNAs are needed per cell—quickly made it the tool of choice for functional genomics (determining what a gene product does and with what other products it interacts) and for drug target discovery and validation. By “knocking down” a gene with RNAi and determining how a cell responds, a researcher can, in the course of only a few days, develop significant insight into the function of the gene and determine whether reducing its expression is likely to be therapeutically useful. But does RNAi have a better chance to succeed as a drug than antisense or ribozymes?

“The fundamental difference favoring RNAi is that we're harnessing an endogenous, natural pathway,” says Nagesh Mahanthappa, Director of Corporate Development at Alnylam Pharmaceuticals in Cambridge, Massachusetts, the second of two major biotech company developing RNAi-based therapy. The exploitation of a pre-existing mechanism, he says, is the main reason RNAi is orders of magnitude more potent than either of the other two types of RNA strategies.

## Delivery, Delivery, Delivery

More potent in the test tube, at least. But stability and delivery are also the major obstacles to successful RNAi therapy, obstacles that are intrinsic to the biochemical nature of RNA itself, as well as the body's defenses against infection with foreign nucleotides. “For the strongest reasons, you can't get away from this,” says Stein. “The problem is that a charged oligonucleotide will not pass through a lipid layer,” which it must do in order to enter a cell. John Rossi, Director of the Department of Molecular Biology at City of Hope Hospital in Duarte, California, who has worked on RNA-based therapies for 15 years, concurs. “The cell doesn't want to take up RNA,” he says, which makes evolutionary sense, since extracellular RNA usually signifies a viral infection. Injected into the bloodstream, unmodified RNA is rapidly excreted by the kidneys or degraded by enzymes.

To solve the problem of cell penetration, most researchers have either complexed the RNA with a lipid or modified the RNA's phosphate backbone to minimize its charge. Mahanthappa thinks the complexing approach is unlikely to be a simple solution, since drug approval would require independent testing of the lipid delivery system as well. Instead, Alnylam is pursuing backbone modification. “Some minimal modification is going to be necessary” to increase cell uptake and to improve stability in the blood stream, Mahanthappa says. “What we have learned from the antisense field is that even without other delivery strategies, when you administer RNA at sufficient doses, if it's stable, it gets taken up by cells.”

“Anything that can be done to increase half-life in circulation would improve delivery,” says Judy Lieberman, a Senior Investigator at the Center for Blood Research and Associate Professor of Pediatrics at Harvard Medical School in Cambridge, Massachusetts. But that may not be the only problem, she cautions. Lieberman's lab recently demonstrated the ability of RNAi to silence expression of the Fas gene in mice, protecting them from fulminant hepatitis. Fas triggers apoptosis, or programmed cell death, in response to a variety of cell insults. In her experiment, Lieberman delivered the RNA by high-pressure injection into the tail. The RNA got to the liver, silenced Fas, and protected the mice from hepatitis. However, a significant fraction of animals died of heart failure, brought on because the injection volume was about 20% of the mouse blood volume. Such a delivery scheme simply will not work in humans. “Delivery to the cell is still an obstacle,” Lieberman explains. “Unless you really focus on how to solve that problem, you're not going to get very far.”

## Unanswered Questions

Even assuming delivery problems can be solved, other questions remain, including that of whether therapeutic levels of RNAi may be toxic. Mahanthappa says, “The conservative answer is we just don't know. The more aggressive answer is there's no reason to think so.” Rossi isn't so sure. “The target of interest may be in normal cells as well as cancer cells,” he says. “That's where you get toxicity.”

But if small RNAs can be delivered to target cells efficiently and without significant toxicity, will they be effective medicines? Usman of Sirna is confident they will be. “If you can get it there, and if it's in one piece, there no doubt in our minds that it will work,” he says. To date, numerous experiments in animal models suggest RNAi can downregulate a variety of target genes effectively. However, there are still two unanswered questions about whether that will translate into effective therapy.

The first is whether RNAi's exquisite specificity is really an advantage beyond the bench. “It's unclear whether highly specific drugs give you a big therapeutic effect,” says Cy Stein. For instance, he says, “most active antitumor medicines have multiple mechanisms of action. The more specific you make it, the less robust the therapeutic activity is likely to be.” Rossi agrees: “Overspecificity has never worked,” he says.

The second question is what effect an excess of RNA from outside the cell will have on the normal function of the RISC, the complex at the heart of the RNAi mechanism. The number of RISCs in the cell is unknown, and one concern is that the amount of RNA needed to have a therapeutic effect may occupy all the available complexes. “We are usurping a natural cell system that is there for some other purpose, for knocking out endogenous gene function,” says Rossi. With the introduction of foreign RNA, will the system continue to perform its normal function as well, or will it become saturated? “That's the big black box in the field,” he says.

## Looking Ahead to the Clinic

Despite the questions and unsolved problems, Sirna, Alnylam, and several other companies are moving ahead to develop RNAi therapy; indeed, some outstanding questions are probably only likely to be answered in the process of therapeutic development. The first applications are likely to be in cancer (targeting out-of-control oncogenes) or viral infection (targeting viral genes). To avoid some of the problems of delivery, initial trials may deliver the RNA by direct injection into the target tissue (for a tumor, for instance) or ex vivo, treating white blood cells infected with HIV, for example.

Having spent a decade trying to develop ribozymes, says Usman, Sirna is prepared for the rough road it faces. “We haven't solved all the problems, but we know how to proceed to work through them. It's no surprise to us—we've seen this movie before.” Usman expects Sirna to file an Investigational New Drug Application with the FDA by the end of 2004 and to have a human clinical trial in progress in 2005. “Without a doubt, there will be an RNAi-based drug within ten years,” Usman predicts.

Stein isn't so sure and thinks that too much is still to be learned about RNAi and the body's reaction to it to be confident that RNA-based therapies will ultimately be successful. “The whole field was founded on the belief it was rational, but there are huge gaps in our knowledge, and so you need a bit of luck to be successful,” he says. “I think people are surprised at how complicated it is, but why should it be any other way? It's an intellectual conceit to think that nature is simple.”

## Abbreviations

dsRNA: double-stranded RNA
FDA: Food and Drug Administration
mRNA: messenger RNA
RISC: RNA-induced silencing complex
RNAi: RNA interference
siRNA: short interfering RNA
